# Supplementary figures and images for: Transcriptome data reveal conserved patterns of fruiting body development and response to heat stress in the mushroom-forming fungus Flammulina filiformis
Source: PLoS One. 2020 Oct 16;15(10):e0239890. doi: 10.1371/journal.pone.0239890 (PMC7567395; doi:10.1371/journal.pone.0239890)

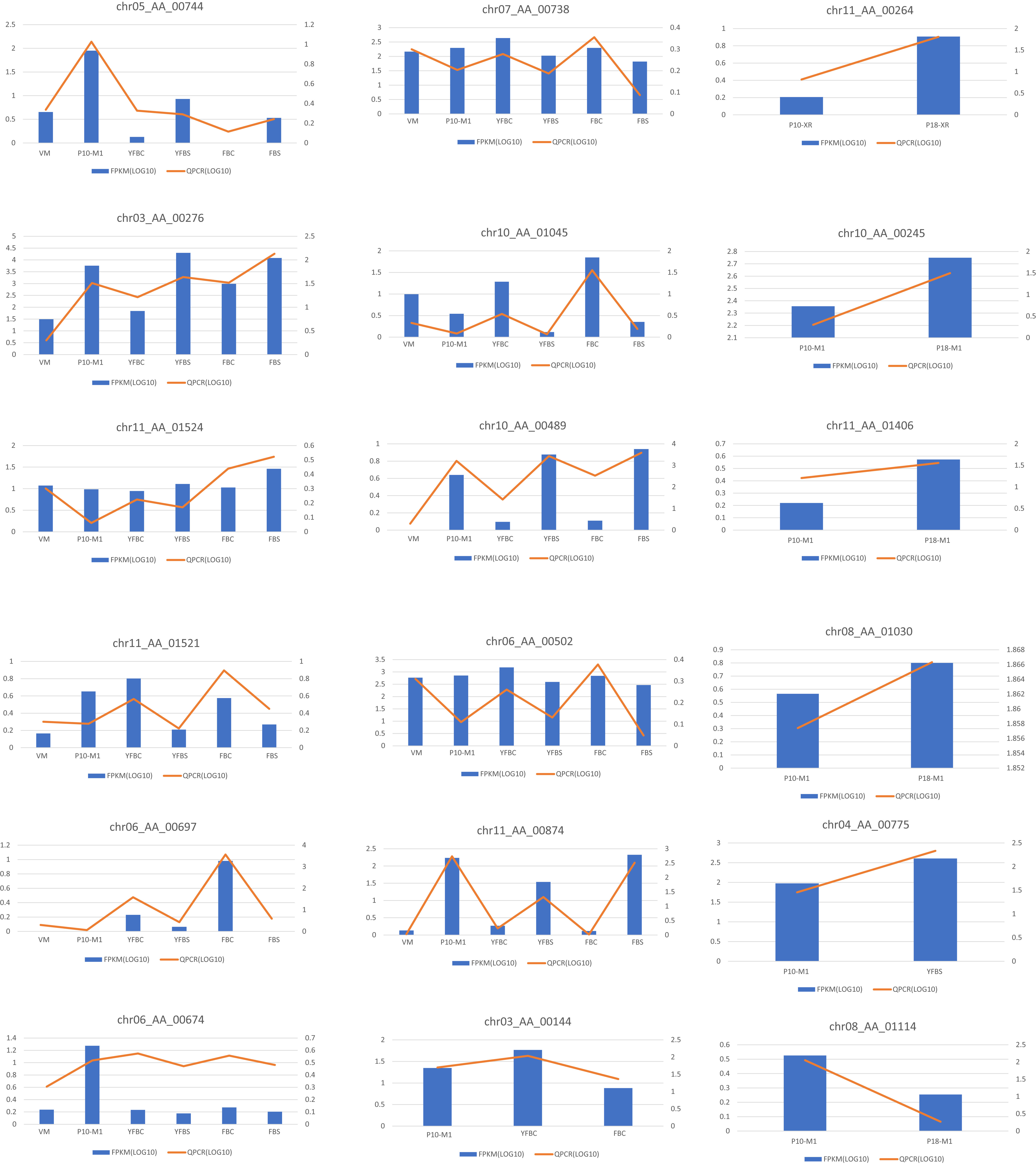

Supplement: S1 Fig — VM, P10-M1, P18-M1, YFBC, YFBS, FBC and FBS correspond to vegetative mycelium, primordium grown at 10°C, primordium grown at 18°C, young fruiting body cap, young fruiting body stipe, fruiting body cap and fruiting body stipe, of strain M1. P10-XR, P18-XR correspond to primordium grown at 10°C and 18°C of strain XR. Bar chart represents the FPKM values (left vertical axis), line chart represents the real-time PCR expression values (right vertical axis). (JPG) [file pone.0239890.s001.jpg]

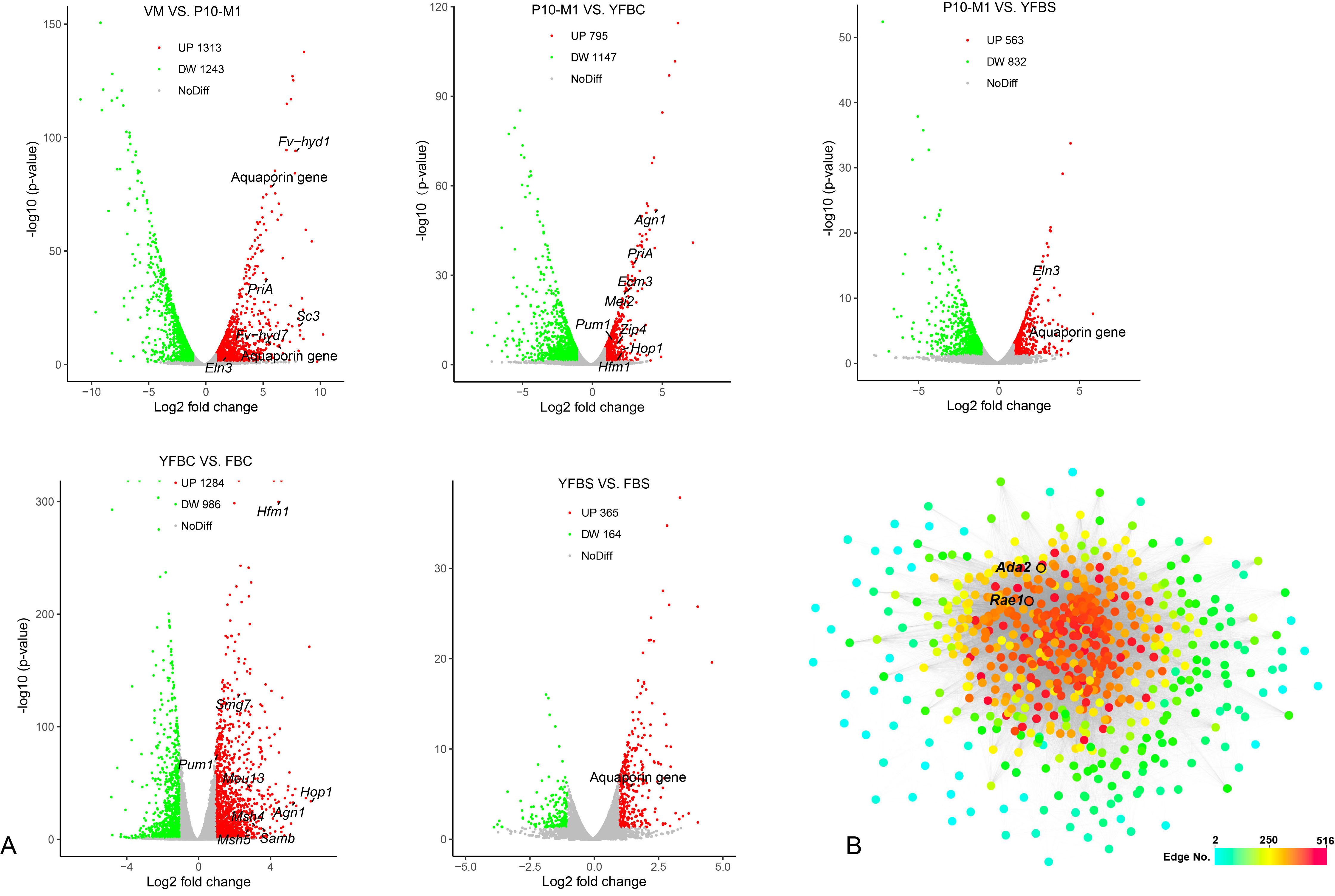

Supplement: S2 Fig — A: Volcano plots of differential expression analysis for each comparison group; B: Gene co-expression network of the young fruiting body cap module (module no. 4 in Fig 2A). The scale bar indicates the number of connections a gene has. (JPG) [file pone.0239890.s002.jpg]

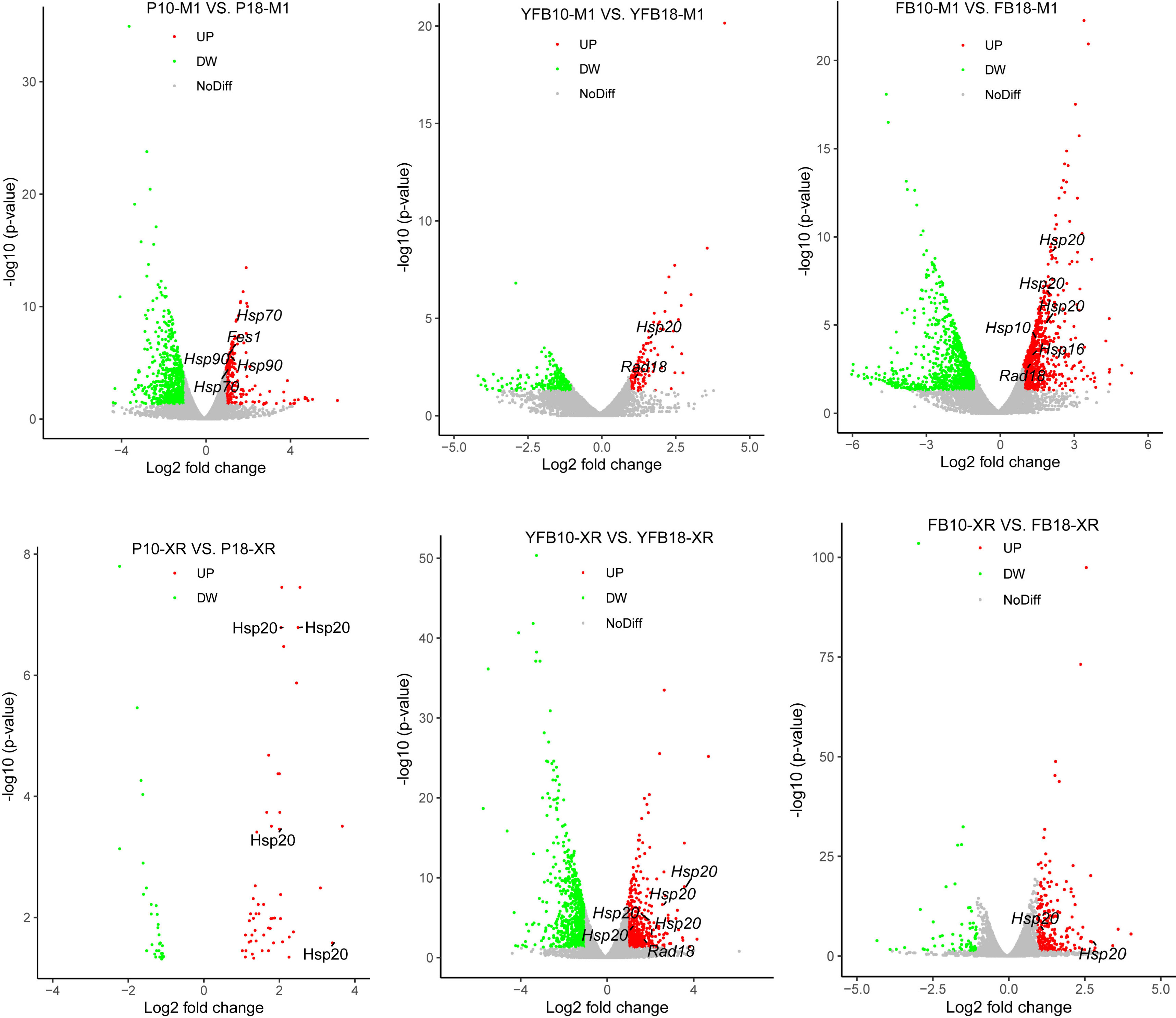

Supplement: S4 Fig — (JPG) [file pone.0239890.s004.jpg]
