# Supplementary material for: Transcriptome data reveal conserved patterns of fruiting body development and response to heat stress in the mushroom-forming fungus Flammulina filiformis
Source: PLoS One. 2020 Oct 16;15(10):e0239890. doi: 10.1371/journal.pone.0239890 (PMC7567395; doi:10.1371/journal.pone.0239890)
Supplement: Attachment — Submitted filename: Response to Reviewers.docx [file pone.0239890.s015.docx]

Dear Editors of PLoS One,

Thank you very much for your email with regard to our manuscript entitled “Transcriptome data reveal conserved patterns of fruiting body development and response to heat stress in the mushroom-forming fungus *Flammulina filiformis*” (PONE-D-20-02460). We found that the critiques, comments and suggestions of the reviewers are very constructive and helpful. We have revised the manuscript according to the comments and suggestions. We really appreciate your kind help. We believe that the revised manuscript is much improved and hope it will be acceptable for publication in PLoS One.

We look forward to your further communications with regard to this manuscript. Below are our responses to the comments raises by the reviewers.

Best wishes,

Xiao-Bin Liu, László G. Nagy, Zhu L. Yang

**Reviewer #1:**

1) Line 198. Read mapping percentages are very low (58.3-71.2%), presumably because the reference assembly is from a different strain. Although this may be hard to avoid in this study, it is still important to realize possible implications. For example, the expression profiles of faster evolving or strain-specific genes will be captured less accurately, since sequence similarity will be less. Please consider adding a few sentences describing this.

Reply: Thank you very much for the suggestion, we add short comments in Lin224-229.

Several Excel files/sheets are provided with the developmentally expressed genes. It would be beneficial to the readers to also include a sheet with the expression of all the genes, whether they are differentially expressed or not. Moreover, as far as I can see no such files are provided for the expression values during the heat/cold experiment (maybe I overlooked it). Please provide the full expression set for those conditions as well.

Reply: Done.

Line 250. Three transcription factors are mentioned. Do these include any of the known regulators of mushroom development that were previously identified in Schizophyllum commune (e.g. c2h2, fst4, etc).

Reply: No, they are not orthologs of the genes identified in previously study in *S. commune*.

Line 211. Please consider introducing the concept of WGCNA here in a few sentences, explaining the goal to the readers of PLOS.

Reply: Done, we add the concept in line 241-242.

Line 249. “this TF”. It seems that this refers to “Gti1/Pac2 family (IPR018608)” mentioned in Line 244 and Table 1. Is this indeed a transcription factor (i.e. DNA-binding) or is it more generally a ‘regulator’?

Reply: It is more generally a regulator. In *Schizosaccharomyces pombe*, the Gti1 protein related to gluconate uptake, the Pac2 related to sexual development. We rephrased the text accordingly.

Line 99. “Previous studies, revealed protein expression patterns”. The comma is not needed here. Moreover, more accurate would be: “gene expression patterns”

Reply: Done, thank you very much.

Line 166. The name of the tool is Hisat.

Reply: Done, thank you very much.

**Reviewer #2:**

- Abstract: The authors speak of >200 reference genomes which were analysed to fish for conserved genes playing a role in mushroom formation. However, they seem not to explain this in detail further down in the manuscript, e.g. in the Material and methods section.

Reply: Thanks for your suggestion. We add it in Material and methods section in Line 203-209.

- Introduction:

o Attention is paid to the two traditional agaric model systems Schizophyllum commune and Coprinopsis cinerea in which advances in molecular genetics techniques allowed to study of fruiting body formation relevant genes. In addition, the authors speak of economically important non-model systems in scientific focus since more recently but do not cite the worldwide cultivated edible mushroom Cyclocybe aegerita (poplar mushroom; formerly Agrocybe aegerita) which in this context, besides its economic importance, seems not only interesting enough to mention for its capability of haploid fruiting without mating and bioactive compound production but, since recently, also allows functional genetics analyses (MGG 294:663-677. <https://doi.org/10.1007/s00438-018-01528-6>; AEM 85:e01549-19. <https://doi.org/10.1128/AEM.01549-19>; Beilstein J. Org. Chem. 2019, 15, 1000–1007. doi:10.3762/bjoc.15.98).

Reply: Thank you very much. We cite this work in introduction section. Line88-91.

- Material & Methods:

o The two investigated strains of F. filiformis do not seem to be available from an internationally accessible strain collection like the Westerdijk Institute (Utrecht, Netherlands) or the ATCC (American Type Culture Collection in Manassas, Virginia, United States). How will the authors guarantee that scientists outside China will not be refused official requests for F. filiformis M1 (CCMSSC04554) and F. filiformis XR for research purposes (independent from collaboration with Chinese scientists) in view of the very restrictive Chinese policy on national bioresources normally resulting in refusal of biomaterial issuance to scientists outside China?

Reply: We are very willing to deposit our strain in relevant collection center. Indeed, one of the strains (*F. filiformis* M1, CCMSSC04554) in our study already deposited in China Center for Mushroom Spawn Standards and Control. We would like to deposit them in other international collection center after the pandemic of COVID-19.

o Furthermore, the reviewer thinks that details should be given on the exact geographic and, if available, the habitat origin of both strains (something like “…strain XR was isolated from a mushroom market…”, line 126, is hardly informative).

Reply: Done. Line136-139.

o The reviewer sees one more shortcoming in this section (RNA-sampling and –sequencing was carried out accurately): the authors should specifically mention how they performed the realtime quantitative PCR (qRT-PCR) assessment of the differential expression of the 22 randomly selected fruiting- and heat-stress correlated genes they mention further down (Fig. S1). This is to include in a small paragraph on qRT-PCR in this section before the study can be accepted for publication in PLoS One.

Reply: Done, we add it in materials and methods section, in Line210-Lin219.

In addition, the figure caption of Fig. S1 should be more informative (the authors should double-check on this also in their other figures): what will “chr11_AA_01521” or “P10-M1” tell the uninformed reader of the study? The reviewer is aware that fruiting stage describing abbreviations like “P10-M1” are spelled out further down in the figure legend of Fig. 2 but explaining these terms in in Fig. S1, too seems adequate to increase readability of the manuscript. Apart from that, there seems to be a discrepancy between the 22 genes checked with qRT-PCR in the manuscript text (line 201) and in the figure legend where it states 18 genes were qRT-PCR-checked (line 695)!

Reply: We add the legend in Fig. S1, the number of the genes involved in qRT-PCR should be 18. Thank you very much.

Terms like “chr11_...” and chr05…” also imply that the reads were potentially mapped to chromosome level of the F. filiformis genome. Could this please be specified somewhere in this section or elsewhere appropriate in the manuscript?

Reply: Yes, the reference genome was assembled in chromosome level, we mentioned it in Line176.

-Results and Discussion:

o Line 268 and Tab. 1: It is mentioned that the flammutoxin-encoding gene is conserved (if this is a typo there "...conversed...", line 268; it should also be "...was..." instead of "...were..." I assume) in 20 Basidiomycota species.

Reply: Done. Thank you very much.

It seems interesting to compare whether other anti-antagonist mushroom compounds like galectins, ribotoxins, or other antimicrobial proteins/peptides are also conserved and expressed (When? In vegetative mycelium or during fruiting?) in F. filiformis? The authors should check on this as well and possibly complement their results with this recommendable checkup.

Reply: Thank you very much for this suggestion. We didn’t find the galectins and ribotoxins coding gene in *F. filiformis* genome. Whereas, we find *Macrolepiota procera* mpl homologous gene in *F. filiformis*. This gene is conserved in Agaricomycetes, and have a conserved expression pattern in fruiting body stage. We discussed its role in line285-lin292.

o Lines 278-280: Maybe the authors should like to shortly emphasise the need for a resilient functional genetics toolbox in F. filiformis that would greatly serve increasing knowledge about this interesting gene? In this context, they may like to discuss which tools (transformation techniques, transformant selection markers, overexpression tools,...) are available for F. filiformis.

Reply: Done, Line296-Line298. Thank you very much.

o One general comment on gene notation: Either the authors follow the widely-applied “basidiomycete code” (wild type gene names written like in yeast code but all letters lowercase; protein names as in yeast code…) or the “yeast code” (wild type gene names written italicised in uppercase letters with a number; proteins non-italicised, first letter uppercase; this code is sometimes applied in Basidiomycota, too, especially when addressing gene names of a certain species like AaeAGT1 for the ribotoxin gene of the poplar mushroom to not get somewhat “inartfully” looking gene names like Aaeagt1). This should be corrected before publication.

Reply: Done. Thank you very much.

**Reviewer #3:**

I enjoyed this study and found their approach to thoughtful. However, I believe that this study, as written, may not appeal to the broad readership of PLOS One. Furthermore, I found their lines of evidence, and their inclusion of supporting literature to be vague. In the first few paragraphs of their Introduction, the authors did not effectively place their study within a broader context. Yet, their Results and Discussion did interpret the implications of previous studies, and how their work contributes to addressing gaps in our knowledge base, as well as contributes valuable data to the genetic underpinnings of fungal fruit body development.

Reply: Thanks for this suggestion. We think we already put our research within a broader context to discuss the complex multicellular evolution from fruiting body development. We also highlighted the importance of our work as uncover the fruiting body development mechanism which can help us to understanding the complex multicellular evolution.

I recommend that the authors re-work both their Introduction and Discussion (Results and Discussion) to place their study within a broader context. Please interpret the cited literature more extensively, and be more specific about the gaps in our knowledge about mushroom development in Agaricomycetes. What triggers fruit body / primordial development? How are these triggers found to elicit reproduction in natural systems? To what extent do mushroom cultivators emulate these processes within the built environment? Why does cultivation require low temperatures of this particular mushroom-- is it for coloration or cluster formation? What is the significance of trehalose biosynthesis genes, and what can it tell us about the mushrooms growing at different temperatures? Why might be expect aquaporins or hydrophobins to arise in these different growing conditions? In general, there needs to be greater rationale for the study and heightened detail about the genes, especially greater background on hypothesized mechanisms. In fact, I would have appreciated more explicitly stated hypotheses, with associated rationale, within the Introduction.

Reply: Thanks for the suggestion, In order to be more specific about the gaps in our knowledge about mushroom development in Agaricomycetes, we interpret the previous work more extensively in introduction part. However, we intend to keep the introduction concise, therefore, we do not go into much detail on some the background of fruiting body induction, rather, we refer to recent reviews on the topic. Line76-line81, line83-line87, line93-line94, line111-line118.

Even before going into great detail with their focal study organism, I recommend that the authors begin with a more general introduction about how organisms (across different trophic levels) respond to environmental stress. Additionally, I recommend that the authors emphasize what gene regulation, as inducible gene expression, could tell us about how bacteria and multicellular organisms, in general, promote their survival in stressful conditions, as well as faced with heat-stress, more specifically. Another approach that I recommend would be to bring in conceptual frameworks, to highlight your intervention within the broader fields, such as an ecological framework, (e.g., Grime’s pyramid, with an emphasis on stress-tolerant strategies), or framing this within an evolutionary context, in terms of selective pressures and eco-physiological and metabolic responses to such pressures. Then, once the rationale is more compelling, I would like the authors to pinpoint their focus on mushrooms, and then F. filiformis.

Reply: Thanks for this suggestion, In order to keep compact and concise of the introduction, we included some general considerations on heat stress response in line117-line118.

I realize that mushroom are important in the ecosystem, as the authors mention at the onset of their manuscript. I certainly appreciated the authors' discussion of Coprinopsis cinerea and Schizophyllum commune, as well as mentioning “ecologically or economically important non-model species.” Yet, why are: Agaricus bisporus, Flammulina filiformis, Armillaria ostoyae, Lentinula edodes and Lentinus tigrinus ecologically important? A little more discussion of this would add some much needed depth to introduce this study.

Reply: Thanks for the suggestion, we revised this in line88-91, with the statement of the saprotrophic fungi, plant pathogen and ectomycorrhizal fungi.

There were minor grammatical errors what interfered with the ease of readability of the manuscript. I recommend that the authors proofread the paper for grammar and flow. Define or limit terms introduced that may be industry specific. Given the broad audience at PLOS One, some terms may require additional clarifying details. For instance (line 135): is "scratched" an industry specific term to mimic disturbance -- leading to an increased investment in reproduction/fruiting to pass on genetic material to progeny?

Reply: The “scratched” is used to describe the physical perturbation of the mycelium surface to stimulate fruiting body development.

Methodologically, this paper had many strengths. However, since the study was not hypothesis driven, it was challenging to determine how the research frames an intervention to fit into a broader context. I recommend that the authors look to cite studies using transcriptomics to examine fungal responses to experimental warming in the field, such as this study on meta-transcriptomics by Romero-Olivares et al. 2019 in Frontiers in Microbiology doi: 10.3389/fmicb.2019.01914, which does an excellent job of framing the stress response trade-offs with decay gene expression. I would recommend a greater discussion of these trade-offs within the sun-section in the Results/Discussion on the CAZymes and F-box genes (L 369-386).

Reply: Thank you very much for the recommended paper. This is a nice paper which investigated the tradeoff in fungal resource allocation under experimental warming treatments. However, in the CAZymes and F-box genes section, we mainly discussed the expression pattern of the CAZyme genes during fruiting body development, since this gene family recently reported not only involved in wood decay, but also related to fruiting body development. Therefore, we think the topic of the paper which reviewer recommends is different from our study, and we do not discuss it in our study.

Although I appreciated the discussion on aquaporins and hydrophobins (line 288-296), I would have liked greater linkages of to function, and specifically a greater discussion comparing and contrasting aquaporins expression within ectomycorrhizal fungi, beyond putative common developmental origins (line 304). Furthermore, I would recommend for these notable genes to be mentioned in the introduction, as well. In the introduction, trehalose synthase genes were mentioned, but then was not detected or discussed in the Discussion. Was this surprising that there were no trehalose synthase genes detected? Please adjust caption for Table 1 (Top 20 and two notable genes…), as that text is unclear. I would have like greater integration of the figures into the manuscipt. Include a list of figures, as the list of supplemental figures are given. When referring to the figure, it is challenging to find the information, required for interpretation. For instance, I recommend that you include legend in fig 1 that describes or identify the color schemes or components of the figures.

Reply: Thank you very much for the comments. We add functional discussion of the aquaporins in Line311-313. In our study, we didn’t detect the differential expression of the trehalose synthase genes. This may be interpreted as the heat treatment in our study was not severe enough, since the trehalose synthase expression often happens under severe heat stress, but may also reflect *Flammulina*-specific traits. We currently don’t have enough data to formulate sounds hypotheses on that.
