# Supplementary material for: Transcriptome data reveal conserved patterns of fruiting body development and response to heat stress in the mushroom-forming fungus Flammulina filiformis
Source: PLoS One. 2020 Oct 16;15(10):e0239890. doi: 10.1371/journal.pone.0239890 (PMC7567395; doi:10.1371/journal.pone.0239890)
Supplement: Attachment — Submitted filename: Response to Reviewers.docx [file pone.0239890.s016.docx]

Dear Editors of PLoS One,

Thank you very much for your email with regard to our manuscript entitled “Transcriptome data reveal conserved patterns of fruiting body development and response to heat stress in the mushroom-forming fungus *Flammulina filiformis*” (PONE-D-20-02460). We carefully revised the critiques, comments and suggestions from the reviewers.

For the strain deposit, we have getting in touch with Westerdijk Fungal Biodiversity Institute, CBS Collection, and got agreement to deposit the two strains (M1 and XR) in CBS. Thus, we posted them by FedEx on 10^th^ July (Track ID: 813740466856). Unfraternally, they were forbidden to export by Customs of China. Therefore, we would like to post them again after the pandemic of COVID-19.

We really appreciate your kind help. We believe that the revised manuscript is much improved and hope it will be acceptable for publication in PLoS One.

We look forward to your further communications with regard to this manuscript. Below are our responses to the comments raises by the reviewers.

Best wishes,

Xiao-Bin Liu, László G. Nagy, Zhu L. Yang

**Reviewer #2:**

#General comments of the reviewer: The reviewer thinks that many of his previous comments have been addressed adequately. The most important exception from this is the still unsatisfying (promise on) availability of the employed F. filiformis strains: The reference by the authors of the deposition of F. filiformis M1 (CCMSSC04554) in the mentioned Chinese culture collection is definitely not enough to ensure availability of the strain for researchers outside China to be able to assess this biological material independently of collaboration with Chinese researchers, for the previously mentioned reason of a restrictive Chinese governmental policy on national resources. In addition, the reviewer assumes that just the promise of depositing both strains of their study (F. filiformis M1 and F. filiformis XR) in a fungal strain collection outside China after the COVID-19 pandemic crisis is settled (when? in 2021?), is certainly not enough to satisfy the PLoS ONE requirements of making research data and material available before the manuscript is accepted for publication.

As a compromise, if deposition of the strains may be easier to conduct (less formalities etc.) at a non-Chinese research institute/lab also working on Flammulina, e.g. the van Peer lab (WUR, Wageningen, NL), such may also be an acceptable minimalist way to ensure that colleagues outside China can access the strains independently. The principal investigator of the van Peer lab has worked on Flammulina in China, so he may be considered (re)liable enough by Chinese authorities.

Until the strain availability issue is dealt with more convincingly, for now, this reviewer refrains from recommending endorsement of the manuscript for publication.

Reply: We have getting in touch with Westerdijk Fungal Biodiversity Institute, CBS Collection, and got agreement to deposit the two strains (M1 and XR) in CBS. Thus, we post them by FedEx on 10^th^ July (Track ID: 813740466856). Unfraternally, they were forbidden to export by Customs of China. Therefore, we would like to post them again after the pandemic of COVID-19.

Specific comments:

- Introduction:

o Attention is paid to the two traditional agaric model systems Schizophyllum commune and Coprinopsis cinerea in which advances in molecular genetics techniques allowed to study of fruiting body formation relevant genes. In addition, the authors speak of economically important non-model systems in scientific focus since more recently but do not cite the worldwide cultivated edible mushroom Cyclocybe aegerita (poplar mushroom; formerly Agrocybe aegerita) which in this context, besides its economic importance, seems not only interesting enough to mention for its capability of haploid fruiting without mating and bioactive compound production but, since recently, also allows functional genetics analyses (MGG 294:663-677. <https://doi.org/10.1007/s00438-018-01528-6>; AEM 85:e01549-19. <https://doi.org/10.1128/AEM.01549-19>; Beilstein J. Org. Chem. 2019, 15, 1000–1007. doi:10.3762/bjoc.15.98).

Reply: Thank you very much. We cite this work in introduction section. Line88-91.

#Reviewer’s comment: Although the authors now mention the fungus, they include a typo when writing its genus name: it must be spelled Cyclocybe aegerita (not Cyclogybe!). In addition, the reviewer notices that the authors only cite the work on the recently established genetic system of this rather important fungal species, but they leave out the equally interesting work on the first Basidiomycota ribotoxin Ageritin published with this species (AEM 85:e01549-19. <https://doi.org/10.1128/AEM.01549-19>). The reviewer kindly asks the authors to also cite this one not least given the potential wide distribution of this basidiomycete ribotoxin among diverse agaricomycetes (including Pleurotus species where pore-forming defense proteins to which category the authors suggest flammutoxin to also belong to), at least when discussing their flammutoxin further down in context of other mushroom toxins (see recommendations below).

By the way: the reviewer also noticed another typo in line 85 when the authors speak about CRISPR/Cas9-methodology established with C. cinerea and S. commune. It must not write “CRISPER/Cas9”…

Reply: Thanks very much, we revised Cyclogybe to Cyclocybe, and CRISPER/Cas9 to CRISPR/Cas9. We cited Tayyrov et al. 2019 in line 294-296.

- Material & Methods:

o The two investigated strains of F. filiformis do not seem to be available from an internationally accessible strain collection like the Westerdijk Institute (Utrecht, Netherlands) or the ATCC (American Type Culture Collection in Manassas, Virginia, United States). How will the authors guarantee that scientists outside China will not be refused official requests for F. filiformis M1 (CCMSSC04554) and F. filiformis XR for research purposes (independent from collaboration with Chinese scientists) in view of the very restrictive Chinese policy on national bioresources normally resulting in refusal of biomaterial issuance to scientists outside China?

Reply: We are very willing to deposit our strain in relevant collection center. Indeed, one of the strains (F. filiformis M1, CCMSSC04554) in our study already deposited in China Center for Mushroom Spawn Standards and Control. We would like to deposit them in other international collection center after the pandemic of COVID-19.

#Reviewer’s comment: As said above, the reviewer assumes that just the promise of depositing both strains of their study (F. filiformis M1 and F. filiformis XR) in a fungal strain collection outside China after the COVID-19 pandemic crisis is settled (when? in 2021?), is certainly not enough to satisfy the PLoS ONE requirements of making research data and material available before the manuscript is accepted for publication suggesting a potential compromise.

#Reviewer’s comment: This previous comment of mine was not addressed in the authors’ point-by-point rebuttal and also not in the revised manuscript!

Reply: Since we mentioned from the title of which this study is focused on the species *F. filiformis*, and we also mentioning the strain M1 and XR belongs to *F. filiformis* in abstract. Therefore, in order to keep the manuscript concise, we didn’t add the species name F. filiformis together with the strain name in other parts of this manuscript.

o The reviewer sees one more shortcoming in this section (RNA-sampling and –sequencing was carried out accurately): the authors should specifically mention how they performed the realtime quantitative PCR (qRT-PCR) assessment of the differential expression of the 22 randomly selected fruiting- and heat-stress correlated genes they mention further down (Fig. S1). This is to include in a small paragraph on qRT-PCR in this section before the study can be accepted for publication in PLoS One.

Reply: Done, we add it in materials and methods section, in Line210-Lin219.

#Reviewer’s comment: Thanks for adding this. Still, please take care to always leave a space between numbers and unit (also throughout in the manuscript => please recheck this everywhere) such as in line 214: “10min”, “5µL” etc. => please correct to: “10 min”, 25 µL” etc.; In addition, please everywhere give “µL” not “uL” as in line 215! Furthermore, a line break in line 220 transferring “Results and Discussion” to the next page would be useful

Reply: Thank you very much, done.

- Results and Discussion:

It seems interesting to compare whether other anti-antagonist mushroom compounds like galectins, ribotoxins, or other antimicrobial proteins/peptides are also conserved and expressed (When? In vegetative mycelium or during fruiting?) in F. filiformis? The authors should check on this as well and possibly complement their results with this recommendable checkup.

Reply: Thank you very much for this suggestion. We didn’t find the galectins and

ribotoxins coding gene in F. filiformis genome. Whereas, we find Macrolepiota procera mpl homologous gene in F. filiformis. This gene is conserved in Agaricomycetes, and have a conserved expression pattern in fruiting body stage. We discussed its role in line285-lin292.

#Reviewer’s comment: Thanks for adding this discussion piece on the Mpl protein of M. procera. I have two friendly calls for correction/modification here: first, the reviewer thinks that the finding by the authors that the F. filiformis genome apparently not contains other relevant mushroom defense proteins like galectin- and ribotoxin-encoding genes is interesting enough to explicitly mention it in the manuscript text shortly (within lines 285 to 292), not least since, for instance, the above mentioned study of Tayyrov et al. (2019; AEM 85:e01549-19) shows that potential basidiomycete ribotoxin orthologs should be quite widely spread among very different agaricomycetes (e.g., among different agarics and boletes, including Pleurotus spp. where, interestingly, pore-forming defense proteins like flammutoxin, assumingly, should also be present in addition to putative ageritin-like ribotoxin genes). Second, there is a typo in line 287: “bodyies” must be changed to “bodies”.

Reply: Thanks for this suggestion. We add the short comments about the ribotoxin in line 294-296. For the typo of “bodyies”, we revised it.
